# A Case Report of Pigmented Purpuric Dermatosis With No Granuloma Formation: A 20‐year Follow‐Up Case Study

**DOI:** 10.1155/crip/5536378

**Published:** 2026-02-10

**Authors:** Yasuhiro Horiuchi

**Affiliations:** ^1^ Division of Dermatology, Tsuruse Orthopedic Clinic, Saitama, Japan

**Keywords:** granuloma formation, hemosiderin, hyperlipidemia, pigmented purpuric dermatosis

## Abstract

In a case of pigmented purpuric dermatosis with hyperlipidemia reported earlier to be persistent for over 20 years, the primary disease was cured by oral rinsing with ozone water; however, the remaining prominent pigmentation was not remarkably improved even with oral administration of vitamin C and laser treatment. A histological examination of the lesion was performed to resolve the difficulty in removing this pigmentation. No granuloma reaction, including giant cells, was observed in the lesion tissue, even after such a long course and significant pigmentation. Granuloma formation in this disease may be a functional problem of the patient′s histiocytes and/or phagocytes rather than its chronic course or excessive hemosiderin deposition. Similarly, hyperlipidemia did not affect granuloma formation.

## 1. Introduction

Pigmented purpuric dermatosis (PPD) [1] is a purpuric skin disease that follows a relatively chronic course and is characterized mainly by perivascular lymphocytic infiltration and pigmentation of hemosiderin deposits due to erythrocyte leakage in the lower extremities. However, its cause and pathophysiology remain unclear. Recently, it was reported that oral rinsing with nano‐ozonated water could be an effective treatment [2], suggesting oral bacteria etiology. Many case reports have indicated that granulomatous reaction is a tissue subtype of a PPD tissue reaction [3–5]. Within the scope of PubMed search, approximately 15 cases have been reported as a histological subtype of this disease. Although some studies have suggested a relationship between granuloma type and hyperlipidemia [6], there are also reports dismissing a clear association with hyperlipidemia in granulomatous PPD cases [7].

In a case of PPD with hyperlipidemia and > 20 years since the onset of the disease, in which abundant hemosiderin pigmentation remained [2], this retrospective follow‐up study examined whether granuloma formation in this disease was a histological subtype; however, no granuloma formation was observed.

## 2. Clinical Summary

We briefly describe the clinical course of PPD in a 75‐year‐old woman, who visited our dermatologic clinic approximately 10 years previously, exhibiting scattered tiny purpuric rashes and pigmented spots and was diagnosed with Schamberg′s disease [1]. She was treated with a mixture of topical steroids and moisturizers without any improvement. Furthermore, approximately 2 years ago, the patient reported that the inflammatory reaction subsided after rinsing the mouth with ozonated water [2]; thus, this disease is possibly caused by oral bacteria. Then, we tried to erase the remaining significant pigmentation with a high‐power short‐pulse Ruby laser several times, but the desired effect was not achieved. The patient had hyperlipidemia, and she had been taking rosuvastatin for hyperlipidemia for more than a year; there was no data then. However, she reported that her purpuric symptoms had been present for several years before taking this medication, suggesting it is not drug‐induced purpura. The total cholesterol occasionally showed a minimally high value of 221 mg/dL (normal range: 130–219 mg/dL) and a normal range low‐density lipoprotein (LDL) value; it was assumed that there was a granulomatous reaction in the pigmented area and a biopsy was performed.

## 3. Pathological Examination and Findings

**Figure 1:**
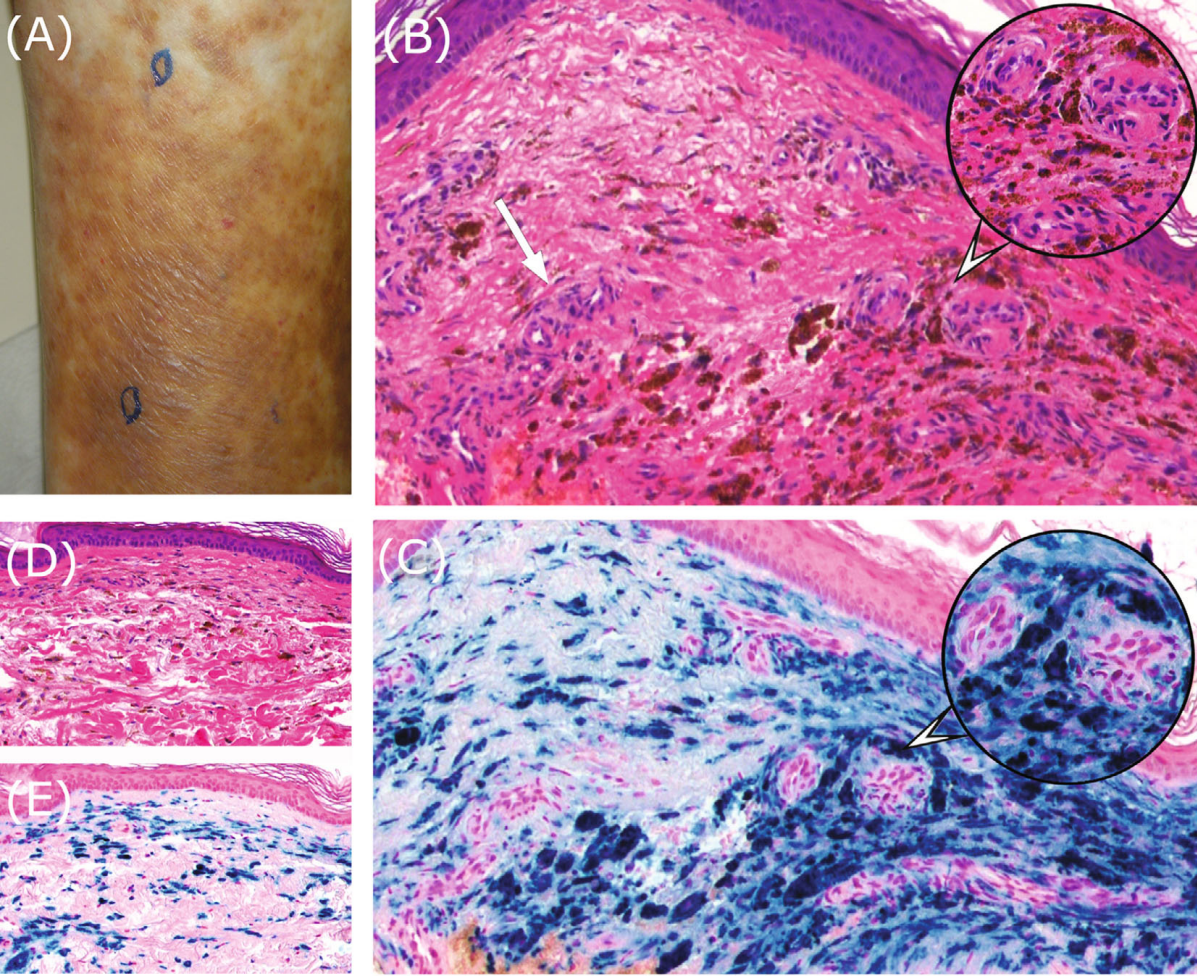
(A) Edematous macular pigmentation on the dorsal Achilles tendon site of the left lower leg without laser treatment and fading pale pigmentation spots on the margin of the pigmented lesion, marked collection sites. (B) Histology of edematous macular pigmentation (hematoxylin–eosin, ×100) shows extensive granular and diffuse pigment mass in the upper dermis, no granulomatous reaction, no perivascular lymphocytic infiltrates, or varying degrees of edema were observed. Note the dilation and meandering of abundant capillary vessels (point with a white arrow), presenting with the strong magnification of the extracted part in a balloon (×200). (C) The finding of Berlin blue stain for hemosiderin iron of edematous macular pigmentation shows extensive hemosiderin pigments and positively stained deposits in the upper dermis, presenting in strong magnification of the extracted part in a balloon (×200). (D) Histology of fading pale pigmentation spots on the margin of the pigmented lesion, notably considerable hemosiderin pigments in the upper dermis (hematoxylin–eosin, ×100), no granuloma features, and no perivascular lymphocytic infiltrates were observed. (E) Finding of Berlin blue stain for hemosiderin iron of fading pale pigmentation spots (×100) on the margin of the pigmented lesion, showing considerable positively stained deposits in the upper dermis.

Skin biopsies were obtained from two sites: marked collection sites from edematous macular pigmentation on the dorsal Achilles tendon area of the left leg and fading pale pigmentation spots on the margin of the pigmented lesion without laser treatment (Figure 1A). Routine hematoxylin–eosin and Berlin blue staining were performed to confirm hemosiderin iron.

In edematous pigmented lesions, extensive pigmentation was observed in the upper dermis with no granuloma reaction, including giant cells, and no perivascular lymphocytic infiltrates. However, varying degrees of manifestations of edema, dilation, capillary vessel distribution (white arrow in Figure 1B), and endothelial cell swelling were exhibited. Berlin blue staining for hemosiderin iron (Figure 1C) confirmed extensive deposits in the upper dermis.

In the areas where the pigmentation had faded or disappeared, considerable pigmentation (Figure 1D), showing scattered Berlin blue–positive pigments (Figure 1E), was observed; however, there was also no lymphocytic infiltration around the blood vessels.

It was also confirmed that the original disease was cured by rinsing the mouth with ozonated water. The only tissue difference between the peripheral improved pigmented lesion and the edematous hyperpigmented area was the amount of deposited hemosiderin.

## 4. Discussion

This patient had already completed the treatment of the primary disease PPD with ozonated water and still had a large amount of hemosiderin pigmentation [2]. More than 20 years had passed since the onset; however, the most important finding of this follow‐up verification is that neither lymphocytic infiltration nor granulomatous reaction was observed in the pigmented lesions.

The distinct histological reactions for PPD include erythrocyte leakage and perivascular lymphocytic infiltrates around microvessels [1], and their features are indistinguishable from other subtypes [8]. Huang et al. [3] reported five distinct histological PPD patterns: lichenoid, perivascular, interface, spongiotic, and granulomatous. A total of 17% of analyzed cases were determined to be lymphocytic vasculitis [3]. Granulomatous was only the least common (3.7%) [3]. Many cases with granuloma‐tissue reaction have been reported [4, 5]. Although studies showed that granuloma‐tissue reaction was related to hyperlipidemia [6], some reports indicated that it was not related [7]. Given that oral rinsing with ozonated water is effective [2], it has been strongly suggested that oral bacteria are the cause. However, the precise etiology is unknown, and the pathological factors are confusing.

Foreign granuloma is thought to be an overreaction or poor digestion of phagocytes, such as macrophages [9] due to foreign substances. In this case, the foreign substance was hemosiderin, and even though the patient′s PPD lesions were resolved with ozone treatment, a large amount of hemosiderin was still deposited. Although granulomas have been reported to disappear after partial excision [10], spontaneous resolution is unlikely in cases in which hemosiderin deposition is prolonged and diffuse, such as in PPD. Nonetheless, it is also true that the excessive hemosiderin deposition in a case persisting over 20 years [2] did not result in granuloma formation. Thus far, it is believed that a granuloma reaction did not occur in the first place. Reports indicated that hemosiderin was present in the tissue of foreign granuloma [11], and it has been reported that giant cells frequently develop as a granulomatous reaction because of hemosiderin in leaking red blood cells [12]. However, it is unclear whether this substance is involved in granuloma formation. Moreover, a binding protein between hemosiderin [13] and ferritin [14] can develop in the presence of inflammation [13], leading to iron‐related pigmentation [15], which is not easily absorbed. In this case, although a large amount of hemosiderin accumulated and remained, no granulomatous reaction occurred, and only unabsorbed pigmentation remained. Phagocytes with sufficient phagocytic function may have been recruited in this patient. So far, it is presumed that neither giant cells nor granulomatous reactions have occurred to eradicate this excessive hemosiderin deposit accumulated during such a long time. Therefore, it has been suggested that the formation of granulomas depends on the genetic predisposition of the patient [16] and species [17], that is, the ability of phagocytes to process foreign substances.

Although hyperlipidemia and granuloma formation remain controversial in this disease, in sulfated polysaccharides, carrageenan injection experiments using hyperlipidemic rabbits have shown that hyperlipidemia is not a direct factor in granuloma development [18]. As pointed out, granuloma formation may be a functional problem of the patient′s phagocytic cells, much less a hyperlipidemia background.

## 5. Conclusion

The difference between the slightly pigmented spots in marginal areas, which clinically improved, and the strongly pigmented macule depended on the amount of hemosiderin deposited. Hyperlipidemia and granulomatous reaction in this disease should be considered only an incidental coexistence. It should be considered that granuloma formation is due to a genetic predisposition to the phagocytic ability of the patient′s histiocytic cells and phagocytes [16]. In cases in which phagocytosis may function adequately, the pigment is gradually digested and excreted. In cases of granulomatous lesions, the patient′s immune response to foreign substances should be verified. It may be necessary to reconsider whether the granuloma‐type reaction, which deviates from the basics of perivascular lymphocytic infiltration and purpura in this disease, is a variant of this disease.

## Ethics Statement

As the study did not include any ethical items, ethical approval was not applied. After obtaining the patient′s consent for the skin test and publication (including the publication of images), the study was conducted in accordance with the Declaration of Helsinki (as revised in 2013).

## Conflicts of Interest

The author declares no conflicts of interest.

## Author Contributions

The author significantly contributed to the manuscript.

## Funding

No funding was received for this manuscript.

## Data Availability

All data is contained in the manuscript.
